# Les amyloses rénales en néphrologie

**DOI:** 10.11604/pamj.2019.34.79.8722

**Published:** 2019-10-11

**Authors:** Gaël Clovis Gassongo Koumou, Daniel Tony Eyeni Sinomono, Siham Merzouk, Nadia Kabbali, Taoufik Harmouch, Mohamed Arrayhani, Tarik Sqalli Houssaini

**Affiliations:** 1 Service de Néphrologie, CHU Hassan II, Fès, Maroc; 2 Equipe de Recherche REIN (Renal Exploration & Investigations in Nephrology), Faculté de Médecine et de Pharmacie, Fès, Maroc; 3 Service d’Anatomopathologie, CHU Hassan II, Fès, Maroc

**Keywords:** Amylose, insuffisance rénale, syndrome néphrotique, Rouge Congo, Amyloidosis, renal failure, nephrotic syndrome, Congo Red dye

## Abstract

Nous nous sommes permis d’étudier les aspects épidémiologiques, cliniques et évolutifs des amyloses rénales, et d’identifier les facteurs de mauvais pronostic. Il s'est agi d'une étude rétrospective, ayant porté sur tous les patients hospitalisés entre janvier 2013 et décembre 2014, pour une amylose rénale. Le diagnostic était confirmé par la ponction-biopsie rénale ou par la biopsie des glandes salivaires accessoires. Vingt-cinq patients étaient colligés dont 17 hommes et huit femmes; âgés en moyenne de 47,2 ± 18 ans. La fréquence hospitalière et le taux d'incidence étaient respectivement de 2,4% et 12,5 cas/an. A l'admission le syndrome néphrotique était retrouvé dans 100% et l'insuffisance rénale dans 68%. La protéinurie était ≥6g/24h dans 60%. Les autres manifestations étaient digestives (n=14), cardiaques (n=10) et l'hypotension artérielle (n=11). Les maladies infectieuses et inflammatoires étaient les principales causes retrouvées (60%). La tuberculose représentait à elle seule 20%. Après un suivi moyen de 219,5 jours, l'insuffisance rénale chronique était notée dans 16 cas (64%) dont 11 cas au stade terminal (44%). Six patients étaient décédés. L'insuffisance rénale au diagnostic, l'aggravation de la fonction rénale et la réhospitalisation étaient corrélées au risque d'insuffisance rénale chronique terminale (p: 0,03-0,04). L'atteinte cardiaque, la réhospitalisation et un débit de protéinurie ≥6g/24h étaient les facteurs associés au risque de décès (p< 0,03). L'insuffisance rénale, l'atteinte cardiaque, le débit de protéinurie ≥6g/24h et la réhospitalisation constituent les principaux facteurs de mauvais pronostic retrouvés dans cette cohorte.

## Abstract (en)

The purpose of this study was to investigate the epidemiological, evolutionary and clinical features of the renal amyloidosis and to identify poor prognostic factors. We conducted a retrospective study focusing on all patients hospitalized for renal amyloidosis between January 2013 and December 2014. The diagnosis was confirmed by renal puncture-biopsy or by biopsy of minor salivary glands. We collected data from 25 patients, 17 men and eight women, with an average age of 47.2 ± 18 years. Hospitalization rate and prevalence were 2.4% and 12.5 cases/year respectively. On admission, nephrotic syndrome was detected in 100% of cases and renal failure in 68% of cases. Proteinuria was ≥6g/24h in 60% of cases. Digestive symptoms (n=14), cardiac symptoms (n=10) and arterial hypotension (n=11) were the other manifestations. Infectious and inflammatory diseases were the main causes found (60%). Tuberculosis alone accounted for 20%. After a mean follow-up period of 219.5 days, chronic renal failure was found in 16 cases (64%), including 11 cases with end-stage disease (44%). Six patients died. Renal insufficiency at the time of diagnosis, the worsening of renal function and readmission were associated with a risk for chronic terminal renal failure (p: 0.03-0.04). Cardiac damage, the readmission and proteinuria ≥6g/24h were factors associated with the risk of mortality (p< 0.03). Renal failure, cardiac damage, proteinuria ≥6g/24h and readmission were the main factors for poor prognosis in this cohort.

## Introduction

Les amyloses constituent un groupe hétérogène de maladies liées au dépôt extracellulaire de protéines fibrillaires insolubles dans les tissus. A ce jour, au moins 21 types sont décrits. Les types amylose amyloid-associated (AA) et amylose immunoglobulinique (AL) sont les plus fréquents [1-7]. L'amylose AA, dite réactionnelle ou secondaire est habituellement associée aux maladies inflammatoires et/ou infectieuses chroniques. L'amylose AL constitue la quasi-totalité des amyloses immunoglobuliniques. Elle est dite primitive et est toujours en rapport avec une population monoclonale de cellules de la lignée B synthétisant une chaîne légère. L'atteinte rénale au cours des amyloses est fréquente et le plus souvent inaugurale. Elle se voit dans plus de 90% des cas au cours des amyloses AA, et dans 50-60% au cours des amyloses AL [2-14]. Les amyloses AL sont les plus fréquentes dans les pays développés alors que les amyloses AA sont plus fréquentes dans les pays en voie de développement [1-11,13,14]. Leur épidémiologie n'est pas bien connue en Afrique. La fréquence varie entre 0,28 et 0,57% en Afrique sub saharienne selon une revue systématique de la littérature [10]. Au Maroc, quelques études ont été menées à ce sujet mais la fréquence demeure mal connue. Les objectifs de ce travail étaient d'étudier les aspects épidémiologiques, cliniques et évolutifs des amyloses rénales; et d'identifier les facteurs de mauvais pronostic.

## Méthodes

Il s'est agi d'une étude rétrospective descriptive et analytique menée dans les Services de Néphrologie-Dialyse-Transplantation Rénale et d'Anatomie Pathologique du Centre Hospitalier Universitaire Hassan II de Fès. Les patients hospitalisés durant la période allant de janvier 2013 à décembre 2014 (deux ans) constituaient notre population d'étude. Etaient inclus, ceux âgés de 16 ans révolus, ayant pour diagnostic de sortie une amylose rénale confirmée à l'histologie sur la ponction-biopsie rénale (PBR) ou la biopsie des glandes salivaires accessoires (BGSA). Les patients ne répondant pas aux critères d'inclusions étaient exclus.

**Critères diagnostiques:** le diagnostic d'amylose rénale était évoqué devant une néphropathie glomérulaire de type syndrome néphrotique avec ou sans insuffisance rénale, associée ou non à un antécédent de maladie inflammatoire ou infectieuse chronique, ou encore à un antécédent de gammapathie monoclonale. La confirmation diagnostique était apportée par la BGSA et / ou la PBR devant la découverte de dépôts amorphes en microscopie optique après coloration par le Rouge Congo, et la constatation d'une biréfringence vert-jaune sur l'analyse en lumière polarisée. Nous avions défini: 1) l'hypertension artérielle par des chiffres de pression artérielle (PA) ≥ 140/90 mm Hg; 2) l'hypotension artérielle par chiffres de PA ≤ 90/60 mm Hg; 3) l'hypotension orthostatique comme étant une diminution de PA systolique d'au moins 20 mm Hg et/ou de la PA diastolique d'au moins 10 mm Hg survenant dans les 3 minutes suivant un passage en position debout; 4) le syndrome néphrotique par l'association d'une protéinurie > 3g/24 heures à une hypoalbuminémie (< 30 g/L). La fonction rénale était évaluée par le dosage de la créatinine plasmatique et l’estimation du début de filtration glomérulaire (DFG) grâce à l’équation de MDRD (modificated of diet in renal disease). L'insuffisance rénale était définie par une créatinine plasmatique >12 mg/L et/ou un DFG < 60 ml/min/1,73m^2^. Elle était dite chronique après un délai > 3 mois. L'évolution était appréciée sur les critères cliniques et biologiques. Elle était dite péjorative en cas de décès et/ou d'installation d'une insuffisance rénale chronique terminale (IRCT). Celle-ci était définie par un DFG < 15 ml/min/1,73m^2^. L'aggravation de la fonction rénale était définie comme étant une augmentation du taux de créatinine d'au moins 25%. Pour chaque patient nous avions étudiés les paramètres anamnestiques (antécédents de maladie infectieuse et/ou inflammatoire chronique; antécédents de gammapathie monoclonale), cliniques, biologiques, évolutifs et pronostiques.

**Plan de recueil des données et échantillonnage**: les patients étaient répertoriés via les registres d'hospitalisation et d'anatomopathologie. Le recueil des données relatives aux différents aspects épidémiologiques, cliniques, biologiques, histologiques, thérapeutiques et évolutifs était fait grâce à une fiche d'enquête pré établie. Les dossiers médicaux et l'historique personnel de chaque patient sur le système intranet « Hosix » avaient servi de sources de données. Ces dernières étaient saisies dans une table Microsoft Excel 2013 préalablement construite en fonction des items de la fiche d'enquête.

**Analyse statistiques des données:** nos données étaient analysées à l'aide du logiciel Epi Info version 7.1.4.0 de juillet 2014. Les analyses étaient d'ordre descriptif et analytique. Les variables quantitatives étaient exprimées en moyenne ± écart-type, les variables qualitatives en pourcentage. La comparaison de deux moyennes était faite avec le test t de Student et la comparaison des pourcentages avec le test de Chi^2^. Une valeur de p inférieure à 0,05 était considérée comme statistiquement significative.

**Patients:** du 1^er^ janvier 2013 au 31 Décembre 2014, 1 382 hospitalisations étaient enregistrées dont 25 pour amylose rénale. Les PBR effectuées à cette période étaient au nombre de 421 dont 22 d'entre elles avaient conclu à une amylose rénale.

## Résultats

La fréquence hospitalière des amyloses rénales était de 2,4%. Celles-ci représentaient 5,2% des PBR. Le taux d'incidence était de 12,5 nouveaux cas par an. Notre effectif comptait 17 hommes et huit femmes, soit un sex-ratio égal à 2,1. L'âge moyen de nos patients était de 47,2 ± 18 ans (extrêmes: 22 et 84). Les tranches d'âges des 25-44 ans et les plus de 55 ans étaient les plus représentatives avec des fréquences respectivement de 44 et 40%. Il s'est agi de nouveaux cas chez 22 patients; d'anciens cas chez trois patients avec une ancienneté respectivement de cinq, sept et 15 ans. Ces trois derniers avaient jusque-là une fonction rénale normale. Aucune histoire familiale d'amylose n'était retrouvée. Tous les patients présentaient un syndrome néphrotique à l'admission. La protéinurie moyenne était de 8,2 ± 5,3 g/24 heures (extrêmes: 3 et 25). Soixante pour cent des patients (n=15) avaient une protéinurie ≥6 g/24 heures. La protidémie et l'albuminémie étaient en moyenne respectivement égale à 45,9 ± 7,8 g/L (extrêmes: 34 et 60) et 15 ± 5,2 g/L (extrêmes: 9 et 30). L'albuminémie était ≤ 20 g/L dans 84% (n=21), et ≤ 15g/L dans 68% (n=17). Le syndrome néphrotique était impur dans 18 cas. Les critères d'impureté étaient: l'insuffisance rénale dans 68% (n=17), l'hypertension artérielle dans 16% (n=4) et l'hématurie microscopique dans 12% (n=3). Les œdèmes des membres inférieurs étaient retrouvés dans 72% (n=18). Ils étaient associés à une ascite dans 10 cas, et à un état d'anasarque dans un cas. La créatinine moyenne des 17 insuffisants rénaux était de 65 ± 50,1 mg/L (extrêmes: 15 et 154). L'insuffisance rénale était aiguë chez 15 patients et chronique chez deux patients âgés de 64 et 70 ans. Ces deux derniers étaient admis respectivement au stade IV et V de leur maladie rénale chronique. L'échographie rénale (disponible chez tous les patients), avait permis d'objectiver des reins de taille normale dans tous les cas. La différenciation cortico-médullaire était bonne dans 23 cas et mauvaise chez les deux patients admis en IRC.

Les manifestations extra rénales (Tableau 1) étaient dominées par les atteintes digestives (n=14), cardiaque (n=10) et l'hypotension artérielle (n=11). L'anémie définie par un taux d'hémoglobine inférieur à 10 g/dl était retrouvée dans 56% (n=14). Les principales co-morbidités retrouvées étaient: l'hypertension artérielle (n=4), le diabète sucré (n=2), l'asthme (n=1) et la sinusite (n=1). Les deux diabétiques étaient âgés de 50 et 58 ans; l'ancienneté de leur diabète était respectivement de 20 ans et de 3 mois. Le premier avait une rétinopathie diabétique avérée. Sa fonction rénale était normale à l'admission. La preuve histologique était apportée par la PBR dans 22 cas. La BGSA avait permis de confirmer le diagnostic d'amylose rénale dans trois cas sur six. Chez les trois autres nous avions eu recours à la PBR pour la certitude diagnostique. Le type d'amylose n'a pu être précisé dans cette étude car l'analyse immuno histochimique n'était pas réalisée. Les maladies infectieuses et inflammatoires chroniques constituaient les causes les plus fréquentes (60% des cas). La tuberculose représentait à elle seule 20% des cas (n=5). Les différentes causes présumées notées dans cette cohorte sont listées dans le Tableau 2. La prise en charge était symptomatique dans tous les cas. Sept patients avaient nécessité un recours à l'hémodialyse aiguë pendant l'hospitalisation. Les indications d'hémodialyse étaient toutes en rapport avec les complications de l'insuffisance rénale: acidose métabolique sévère (n=4), hyperkaliémie (n=3), hyperurémie symptomatique (n=3) et œdème aigu du poumon (n=1). Les trois patients myélomateux avaient bénéficiés par ailleurs des cures de chimiothérapie.

**Tableau 1:** Répartition des patients selon les manifestations extra rénales

Variables	Effectif
**Manifestations digestives**	**14**
Splénomégalie isolée	2
Hépato-splénomégalie	2
Vomissements	4
Diarrhée et vomissements	2
Macroglossie	1
Cholestase	2
Cholestase associée à une cytolyse	1
**Manifestations cardiaques**	**10**
**Insuffisance cardiaque globale**	2
**ECG disponible chez 10 malades**	
Microvoltage	7
Hypertrophie du ventricule gauche*	1
**Echocardiographie transthoracique disponible chez 10 patients**	
Fraction d’éjection altérée**	1
Hypertrophie du ventricule gauche ***	3
Ventricule droit dilaté	2
Veine cave inférieure dilatée**	2
Veine cave inférieure compliante et non dilatée	8
Pressions de remplissage élevées	2
Décollement péricardique	3
Epanchement péricardique circonférentiel	2
**Manifestations pleuropulmonaires**	**8**
Pleurésie	4
Séquelles de tuberculose pulmonaires	5

**Légende.** ECG: électrocardiogramme

* : patient hypertendu

** : patient en insuffisance cardiaque globale

*** : un patient était hypertendu et présentait une insuffisance cardiaque globale.

**Tableau 2:** Les différentes causes présumées d’amylose rénale et leur ancienneté

Variables	Effectif	Ancienneté
Maladies inflammatoires chroniques	8	
Polyarthrite rhumatoïde	2	10 ans; 20 ans
Rhumatisme articulaire aigu	2	8 ans; 20 ans
Fièvre méditeranéenne familiale	1	Inconnue
Lupus érythémateux aigu disséminé	1	8 ans
Maladie de Behcet	1	10 ans
Maladie de Crohn	1	7 ans
Maladies infectieuses et surinfections	7	
Tuberculose	5	1 an; 1 an; 19 ans; 20 ans; 40 ans
Dilatation des bronches	2	28 ans; 30 ans
Myélome multiple	3	
Myélome multiple stade III	2	Découverte concomitante
Myélome multiple stade I	1	2 ans
Causes indéterminée	7	

A l'issue de leur première hospitalisation, huit patients avaient nécessité ultérieurement une réhospitalisation. La fréquence des réhospitalisations était d'une fois chez sept patients et de cinq fois chez un patient. Le Tableau 3 passe en revue les différents motifs ainsi que la fréquence des réhospitalisations. L'évolution de la fonction rénale de nos patients tout au long de leur suivi s'était soldée par la survenue d'une insuffisance rénale (IR) dans quatre cas, l'aggravation de l'IR préexistante dans sept cas (Tableau 4). De nos 25 patients, six étaient perdus de vue. Les 19 restants avaient eu un suivi moyen de 219,5 ± 200 jours (extrêmes: 21 et 660 jours). La durée de suivi était de 21 à 90 jours chez trois patients. Au dernier suivi trois patients gardaient une fonction rénale normale, 16 patients étaient en insuffisance rénale chronique (IRC). Celle-ci était de stade V dans 11 cas, stade IV dans trois cas et de stade III dans deux cas. Le taux de mortalité était de 24% (n=6). Les causes de décès étaient, le choc septique (n=4) et le choc cardiogénique (n=2). Cinq des six patients décédés avaient eu chacun une réhospitalisation. L'insuffisance rénale à l'admission (p= 0,04), l'aggravation secondaire de la fonction rénale (p= 0,03) et la réhospitalisation (p= 0,02) constituaient les principaux facteurs corrélés au risque de survenue de l'IRCT après analyse univariée. L'atteinte cardiaque, la réhospitalisation et un débit de protéinurie ≥6 g/24 heures étaient corrélés au risque de décès après analyse univariée (p< 0,03).

**Tableau 3:** Motifs et fréquence des réhospitalisations

Variables	Nombre de réhospitalisation	Nombre de patients
**Aggravation de la fonction rénale**	**8**	**8**
Sepsis sur infection digestive	3	3
Insuffisance cardiaque globale	2	2
Déshydratation extra cellulaire	1	1
Altération de l’état général	1	1
Inflation hydrique sans insuffisance cardiaque	1	1
Chimiothérapie du myélome multiple*	**4**	**1**

**Légende**:

* les réhospitalisations pour chimiothérapie étaient relatives à une patiente âgée de 64 ans traitée par le protocole MDT (Melphalan-Dexamethazone- Thalidomide). Elle était suivie depuis deux années pour un myélome multiple stade IA de Salmon et Durie, traitée initialement par le protocole VAD (Vincristine-Adriblastine-Dexamethazone) sans succès. Elle était admise pour la prise en charge d’une insuffisance rénale sévère.

**Tableau 4:** Répartition des patients selon l’évolution de la fonction rénale (créatinine)

Variables	Effectif
**Fonction rénale normale au diagnostic**	**8**
Normale pendant le suivi	3
Survenue d’une IR pendant le suivi*	4
Perdu de vue	1
**Fonction rénale altérée au diagnostic**	**17**
Insuffisance rénale chronique au diagnostic	2
Insuffisance rénale stable pendant le suivi	1
Baisse de la créatinine sans sa normalisation	2
Aggravation pendant le suivi	7
Perdu de vue	5
**Total**	**25**

**Légende**. IR: insuffisance rénale.

* l’IR était survenue après un délai de: un mois (n=1), trois mois (n=2) et cinq mois (n=1).

## Discussion

A ce jour, plusieurs types d'amyloses sont décrits. Les amyloses systémiques AA et AL sont de loin les types les plus fréquents [3-6,8]. L'incidence et la fréquence de ces maladies sont mal connues et demeurent très variables d'un pays à un autre et d'une région à une autre. L'incidence des amyloses généralisée (AA et AL) fondées sur les certificats de décès serait de 13,3 par million et par an en Hollande [15]. Nous rapportons une fréquence hospitalière de 2,4%, et un taux d'incidence de seulement 12,5 nouveaux cas par an. Nos patients sont âgés en moyenne de 47,2 ans avec une nette prédominance masculine, deux hommes pour une femme (p<0,001). Les tranches d'âges de 25-44 ans et des plus de 55 ans sont les plus représentatives. Nos constatations rejoignent celles des travaux de plusieurs auteurs selon lesquels l'âge moyen au diagnostic d'amylose varie entre 33 et 56 ans avec une predominance masculine [5,6,8-10,13,14]. Selon ces mêmes auteurs, il existe une prédominance des cas d'amyloses entre 50 et 70 ans. Ces constatations s'expliqueraient à travers notre étude, par la prépondérance des maladies infectieuses et inflammatoires chroniques comme étiologies d'amylose rénale dans notre cohorte. En effet les maladies infectieuses pouvant se voir à tout âge alors que les maladies inflammatoires chroniques sont l'apanage du sujet âgé. La fréquence histologique des amyloses rénales était de 5,2% (22/421) sur l'ensemble des PBR. Bziz A *et al.* rapportent une fréquence similaire (30/600) dans une récente étude prospective marocaine [14].

L'atteinte rénale, très fréquente et souvent inaugurale, s'observe dans plus de 90% des cas au cours des amyloses AA et dans 50-60% au cours des amyloses AL [2-14, 16]. Elle se caractérise par une protéinurie évolutive faisant siège à un syndrome néphrotique persistant malgré le degré d'IRC. Cette dernière peut tout aussi être inaugurale ou apparaître secondairement. L'atteinte rénale est constante dans notre cohorte, caractérisée par un syndrome néphrotique dans tous les cas. Bziz *et al* rapportent une fréquence de 94% pour le syndrome néphrotique [14]. L'insuffisance rénale retrouvée dans 68% (n=17) à l'admission dans ce travail corrobore avec les données de plusieurs études où sa fréquence varie entre 20 et 73% sans distinction de type d'amylose [14,17,18]. Le syndrome œdémateux est rapporté dans 50-100% [5,8,9,13] dans la littérature et dans 72% (n = 18 cas) dans notre étude. L'atteinte cardiaque exceptionnelle au cours des amyloses AA, elle est surtout l'apanage des amyloses AL où sa fréquence varie de 60 à 80% [3,6,14,16,19]. Elle réalise un tableau de cardiomyopathie restrictive dont l'amylose AL représente la première cause. L'insuffisance cardiaque se voit dans 25-65% des cas. La principale anomalie électrocardiographique est le microvoltage (50-80%). L'échocardiographie permet d'objectiver d'une part l'aspect de cardiomyopathie restrictive caractérisée par l'hypertrophie des ventricules prédominant au niveau du septum interventriculaire et d'autre part l'aspect « granité » diffus du myocarde, pathognomonique de l'amylose. L'épanchement péricardique se voit dans 58% des cas. L'atteinte cardiaque retrouvée dans notre série est compatible avec la littérature. Mais seulement nous n'avons pas noté de cardiopathie restrictive avérée ni d'aspect « granité » diffus du myocarde à l'échocardiographie. L'atteinte digestive se voit aussi bien au cours des amyloses AA qu'AL. Les manifestations sont fréquentes mais aspécifiques. Leur fréquence varie entre 70-80% des cas selon les auteurs [6,8,13,15,20-22]. Bien qu'aucun examen histologique du tube digestif n'ait été réalisé dans ce travail, les manifestations digestives recensées sont les mêmes que celles listées dans la littérature. La confirmation diagnostique de l'amylose repose sur l'analyse en microscopie optique d'une biopsie après coloration par le Rouge Congo et lue avec un microscope à lumière polarisée. Le diagnostic est confirmé devant la présence d'une biréfringéance vert-jaune. Cela est le cas chez tous nos patients. La sensibilité de la BGSA pour le diagnostic de l'amylose est de 50% dans ce travail et respectivement de 81 et 86% d'après Fatihi E [9] et Hachulla E [23] dans des études consacrées à l'évaluation de la sensibilité de la BGSA.

Depuis l'avènement des antibiotiques, l'amélioration de la qualité de vie et l'accès aux soins pour tous dans les pays développés, l'épidémiologie des causes d'amyloses dans ces pays s'est distinguée de celle des pays en voie de développement. Les maladies inflammatoires chroniques constituent la première cause des amyloses AA en occident. Cependant dans les pays en voie de développement les causes infectieuses demeurent toujours au premier rang, dominées par la tuberculose qui sévit encore de façon endémique. La tuberculose est en tête des différentes causes dans cette série (20%) et dans la récente série de Bziz A *et al* (33%) [14]. Contrairement aux pays développés où l'amylose AL est plus fréquente, dans les pays en voie de développement c'est plutôt l'amylose AA qui est prédominante [1,5,7,10]. Nous n'avons pas pu réaliser l'étude immunoistochimique. Les antécédents de maladies infectieuses et inflammatoires chroniques étaient retrouvés dans 60% (n=15) suggérant une amylose AA ce qui corroborerait aux données de la littérature. Il est admis que l'IR et l'atteinte cardiaque constituent les facteurs de mauvais pronostic au cours des amyloses [1-3,11,19]. L'IR est un facteur péjoratif indépendamment du pronostic vital des amyloses systémiques. L'atteinte cardiaque est étroitement associée au risque de décès. L'évolution naturelle de la néphropathie amyloïde se fait inéluctablement vers l'IRCT. Il en a été ainsi dans cette cohorte où plus de la moitié de patients ont vu apparaître une IRCT au cours du suivi. Le taux de mortalité est de 24% (n=6) et, les causes de décès retrouvées sont: le sepsis (n=4) et le choc cardiogénique (n=2). L'atteinte cardiaque classiquement associée au risque de décès, l'est également dans ce travail au même titre que la réhospitalisation et un débit de protéinurie ≥6 g/24 heures. L'implication de la protéinurie des 24 heures peut être expliquée par les complications infectieuses en rapport avec le syndrome néphrotique. Celui-ci est à l'origine d'un déficit immunitaire responsable de la susceptibilité aux infections. Le déficit immunitaire est dû à la fuite urinaire des immunoglobulines en occurrence les IgG [17,18]. La réhospitalisation était associée à la fois au risque de décès et d'évolutivité vers l'IRCT. Dans les deux cas, ce fait est expliqué par les motifs de réhospitalisation, qui sont dominés par le sepsis et l'insuffisance cardiaque dans ce travail. Ces deux pathologies sont responsables d'une altération de la fonction rénale par le biais d'un syndrome cardio rénal types 1 et 2 pour l'insuffisance cardiaque et de type 5 pour le sepsis.

## Conclusion

Les facteurs de mauvais pronostic retrouvés dans cette série sont l'insuffisance rénale, l'atteinte cardiaque, un débit de protéinurie ≥ 6 g/24 heures et la réhospitalisation. Ce dernier facteur traduit la gravité clinique des patients ayant fait l'objet d'une réhospitalisation ultérieure. En effet ceux-ci étaient réadmis majoritairement pour un sepsis sévère et une insuffisance cardiaque globale, deux pathologies responsables d'une altération de la fonction rénale par le biais d'un syndrome cardio rénal.

### État des connaissances actuelles sur le sujet

- L'épidémiologie des amyloses dans les pays développés se distingue de celle des pays en voie de développement;
- Les amyloses AA sont devenues rares dans les pays développés, mais elles occupent la première place dans les pays en voie de développement où la tuberculose sévit encore de façon endémique;
- L'atteinte rénale et l'atteinte cardiaque sont des facteurs péjoratifs au cours de l'évolution des amyloses. L'atteinte rénale est quasi constante et évolue inéluctablement vers l'insuffisance rénale chronique terminale alors que l'atteinte cardiaque est associée au risque de décès.

### Contribution de notre étude à la connaissance

- Les facteurs de mauvais pronostiques classiques ont été retrouvés dans cette étude;
- Par ailleurs un débit de protéinurie ≥ 6 g/24 heures et les hospitalisations itératives ont été associés au risque de décès dans ce travail.

## Conflits d’intérêts

Les auteurs ne déclarent aucun conflit d'intérêts.
